# Integrative analysis highlights molecular and immune responses of tick *Amblyomma americanum* to *Escherichia coli* challenge

**DOI:** 10.3389/fcimb.2023.1236785

**Published:** 2023-07-31

**Authors:** Bo Lyu, Jingjing Li, Brigid Niemeyer, Deborah M. Anderson, Brenda Beerntsen, Qisheng Song

**Affiliations:** ^1^ Division of Plant Science and Technology, University of Missouri, Columbia, MO, United States; ^2^ Department of Veterinary Pathobiology, University of Missouri, Columbia, MO, United States

**Keywords:** *Amblyomma americanum*, innate immune system, gram-negative bacterium, transcriptome, gene regulation

## Abstract

Ticks are ectoparasites that can transmit various pathogens capable of causing life-threatening illnesses in people and animals, making them a severe public health threat. Understanding how ticks respond to bacterial infection is crucial for deciphering their immune defense mechanisms and identifying potential targets for controlling tick-borne diseases. In this study, an in-depth transcriptome analysis was used to investigate the molecular and immune responses of *Amblyomma americanum* to infection caused by the microinjection of *Escherichia coli*. With an abundance of differentially expressed genes discovered at different times, the analysis demonstrated significant changes in gene expression profiles in response to *E. coli* challenge. Notably, we found alterations in crucial immune markers, including the antimicrobial peptides defensin and microplusin, suggesting they may play an essential role in the innate immune response. Furthermore, KEGG analysis showed that following *E. coli* exposure, a number of key enzymes, including lysosomal alpha-glucosidase, fibroblast growth factor, legumain, apoptotic protease-activating factor, etc., were altered, impacting the activity of the lysosome, mitogen-activated protein kinase, antigen processing and presentation, bacterial invasion, apoptosis, and the Toll and immune deficiency pathways. In addition to the transcriptome analysis, we constructed protein interaction networks to elucidate the molecular interactions underlying the tick’s response to *E. coli* challenge. Hub genes were identified, and their functional enrichment provided insights into the regulation of cytoskeleton rearrangement, apoptotic processes, and kinase activity that may occur in infected cells. Collectively, the findings shed light on the potential immune responses in *A. americanum* that control *E. coli* infection.

## Introduction

The prevalence of ticks and the diseases they transmit has raised serious concerns throughout the world (Randolph, 2008; Estrada-Peña, 2015). These small arachnids, commonly found in grassy and wooded areas, have gained attention due to their ability to transmit a wide range of pathogens to humans and animals. As ectoparasites, ticks have a complex life cycle with four stages: egg, larva, nymph, and adult (Apanaskevich et al., 2014). During each stage of larval to adult development, they require a blood meal to progress to the next stage. Ticks are known for their ability to adapt to various hosts, including mammals, birds, and reptiles, and they are capable of transmitting a variety of bacterial, viral, and parasitic pathogens while feeding that cause illnesses. Transmission of pathogens by ticks occurs as a consequence of a complex cycle whereby the microbe must survive immune responses in the midgut and hemocoel while they transit to the salivary glands. Within salivary glands, some pathogens colonize and grow, facilitating their subsequent transmission to a mammalian host. Furthermore, bacterial pathogens, including *Rickettsia spp*, and *Borrelia spp*, as well as viruses are transovarially and vertically transmitted. These collective characteristics are suggestive that ticks may lack fundamental pathways for responding effectively to infection and/or that pathogens have evolved a complex relationship with their tick hosts that includes immune modulation and evasion.

At least 116 species of ticks have been shown to be associated with transmitting arthropod-borne diseases to animals and humans globally (Brites-Neto et al., 2015). In the United States, *Amblyomma americanum* has become a significant vector of morbid human diseases, including spotted fevers, ehrlichiosis, as well as the hemorrhagic fevers Powassen and Heartland, due to its substantial geographic expansion over the past several decades (Sonenshine, 2018). Until recently, our understanding of mechanisms that underlie the transmission of pathogens by *A. americanum* or any other tick species were limited by a severe lack of whole genome sequences as well as technologies for knocking down gene expression in ticks that allow for experimental validation of biologically relevant pathways. Recent advances now allow for the analysis of global gene expression and we can now identify transcriptional networks that define innate immune responses as well as cellular responses to invasion by microbial pathogens.

Ticks can transmit many diseases, such as Lyme disease, babesiosis, ehrlichiosis, Rocky Mountain Spotted Fever, anaplasmosis, Southern Tick-Associated Rash Illness, Tick-Borne Relapsing Fever, and tularemia. For instance, in the case of Lyme disease, the tick-borne bacterium *Borrelia burgdorferi* possesses mechanisms to evade the host’s immune system by altering its surface proteins, antigenic variation, and immune modulatory factors. This allows the bacterium to establish persistent infection and cause the characteristic symptoms of Lyme disease (Brites-Neto et al., 2015; Nathavitharana and Mitty, 2015; Sparagano et al., 2022). Interestingly, ticks have developed remarkable strategies to coexist with these diverse disease-causing pathogen challenges. Hence, understanding the biology and immunology of ticks and adopting preventive measures are crucial in mitigating the risk caused by ticks.

Ticks are remarkable creatures with a unique ability to survive and thrive in various environments. Their resilience is due to their adaptable physiology and innate immune system, which serves as the first line of defense against invading pathogens (Kopáček et al., 2010; Torina et al., 2020). Understanding the complexities of innate immunity will help us better understand the survival techniques used by ticks as well as provide information on potential defense mechanisms against disease-causing pathogens transmitted by ticks. Compared to the immune systems of vertebrates, the immune systems of ticks and other invertebrates are more primitive, exhibiting solely innate immunological responses (Sonenshine and Macaluso, 2017; Fogaça et al., 2021). Ticks have a wide variety of pattern recognition receptors (PRRs), including lectins, scavenger receptors, and Toll-like receptors (TLRs) (Grubhoffer and Jindrák, 1998; Aung et al., 2011; Fogaça et al., 2021). These receptors identify pathogen-associated molecular patterns (PAMPs) and trigger specific immune responses. As part of their immune response, tick cells synthesize and release a variety of antimicrobial peptides (AMPs) when they identify infection. Defensin, microplusin, and ixosin are examples of tick-derived AMPs with potent antimicrobial activity against bacteria, fungi, and some parasites (Yu et al., 2006; Martins et al., 2019; Li et al., 2021). The formation of reactive oxygen species, complement-like molecules, and phagocytosis are also thought to play a significant role in preventing infection in ticks (Kopáček et al., 2010). The immune deficiency (IMD), Janus kinase/signal transducer and activator of transcription (JAK/STAT), and Toll pathways are crucial for the control of bacterial (e.g., *B. burgdoferi*, *Anaplasma marginale*, and *A. phagocytophilum* infections), viral, and parasitic challenges (Smith et al., 2016; McClure Carroll et al., 2019; Fogaça et al., 2021). Therefore, understanding the complexities of tick innate immunity opens possibilities for cutting-edge methods of preventing tick-borne infections and protecting public health.

Our understanding of arthropod immunity primarily stems from research conducted on *Drosophila* and other insects. However, solely focusing on establishing a direct link between immune signaling and effector specificity in ticks might overlook crucial and novel mechanisms within the tick immune system (Fogaça et al., 2021). Recent studies have proposed that ticks exhibit diverse types of effectors involved in immune signaling pathway activation and uncanonical IMD pathways (Narasimhan et al., 2014; McClure Carroll et al., 2019). Additionally, it is crucial to acknowledge that *Drosophila*, as a non-vector model, cannot fully replicate all aspects of pathogen-vector interactions. Consequently, exploring the tick immune system and its interactions with microorganisms represents a vast and unexplored area deserving further investigation.

This study used integrative RNA-seq analysis to investigate the molecular and innate immune responses of *Amblyomma americanum*, commonly known as the lone star tick, upon treatment with the gram-negative bacterium *Escherichia coli*. *A. americanum* is a common tick species found in the southeastern and eastern regions of the United States. Known for its aggressive feeding behavior, the lone star tick poses a potential public health concern due to its ability to transmit various pathogens, including *Ehrlichia, Anaplasma*, and *Rickettsia*. *E. coli* is a well-characterized and widely studied bacterium, offering a simpler and more controlled experimental system compared to working with a complex tick-borne pathogen. This allows researchers to dissect and understand fundamental immune mechanisms without the confounding factors associated with natural pathogens. Before investigating the immune response to tick-borne pathogens, it is often necessary to establish a baseline understanding of the tick’s immune system. Using a model organism like *E. coli* allows researchers to first characterize the general immune response pathways, gene expression patterns, and immune-related factors in ticks, allowing for a better understanding of how tick-borne pathogens overcome tick immune responses to transmit pathogens in animals. Here, we hypothesized that when exposed to *E. coli*, *A. americanum* would exhibit specific molecular responses as part of its immune defense. These responses may alter immune-related genes involved in recognition, signaling, and antimicrobial defense. These responses are predicted to be an integral part of *A. americanum*’s defense strategy against bacterial pathogens, contributing to its ability to survive and potentially transmit pathogens to its hosts. By investigating these molecular and immune responses, we aim to gain insights into the complex interactions in the tick innate immune systems, ultimately enhancing our understanding of tick immune defenses and their implications for tick-borne disease transmission.

## Methods

### Tick materials and sample collection

We acquired unfed adult female *A. americanum* tick samples from Boehringer Ingelheim Vetmedica, Inc. (Fulton, Missouri, USA). Ticks were all laboratory reared on contained animal systems approved by site IACUC and stored in clean vials between required feedings. Containers were cleaned when they became soiled. Before processing, the ticks were all decontaminated in the lab using an ethanol rinse three times. The tick samples were maintained in a well-regulated laboratory setting (27°C, 80–90% relative humidity, and a 16:8 h light:dark cycle). Tick samples were injected with the common gram-negative bacterium *E. coli* (50 nL, 10^7^) (DH5α strain, Thermo Fisher Scientific Inc., USA) for 3 h (E3), 6 h (E6), 12 h (E12), and 24 h (E24), respectively, to examine the reactions during gram-negative bacterial challenge. Preliminary research revealed that, in comparison to other dosages, this particular dose (50 nL, 10^7^) increased the expression of two AMPs following *E. coli* treatment (**Figure S1**). The control groups received the same volume of PBS (50 nL, 10 mM, pH 7.2), a phosphate buffer saline solution. A Nanoject II AutoNanoliter Injector (Drummond Scientific Co., Broomall, PA, USA) outfitted with a 3.5-inch glass capillary tube pulled by a needle puller (Model P-2000, Sutter Instruments Co., Novato, CA, USA) was used to inject *E. coli* and PBS solution into the ventral side near the anus of ticks under a Leica M205 C stereomicroscope (Leica Microsystems, Wetzlar, Germany). At each time point, whole tick samples were homogenized in 500 μL of TRIzol using a homogenizer and refrigerated at -80°C. For RNA-seq expression profiling, a total of 72 ticks were gathered and grouped into eight groups (three biological replicates, each with three ticks).

### cDNA library establishment and assembly

The RNA sample preparations used 1.5 μg of RNA as input material for each sample. The RNA features (quantity and quality) were detected using nanodrop and agarose gel electrophoresis. The RNA integrity was measured using the RNA Nano 6000 Assay Kit of the Agilent Bioanalyzer 2100 system (Agilent Technologies, CA, USA). The sequencing libraries were obtained using the NEBNext® UltraTM RNA Library Prep Kit for Illumina® (NEB, USA) in accordance with the manufacturer’s instructions. The mRNA was extracted from total RNA using poly-T oligo-attached magnetic beads. Divalent cations were used to carry out fragmentation at a high temperature in NEBNext First Strand Synthesis Reaction Buffer (5X). Using M-MuLV Reverse Transcriptase (RNase H-) and random hexamer primers, first-strand cDNA was created. Then, second strand cDNA synthesis was carried out utilizing DNA Polymerase I and RNase H, and the remaining overhangs were turned into blunt ends using exonuclease/polymerase. NEBNext Adaptors with a hairpin loop structure were ligated to prepare for hybridization after the 3’ ends of DNA fragments were adenylated. The library fragments were purified using the AMPure XP technology (Beckman Coulter, Beverly, USA) in order to identify cDNA fragments that were preferably 250–300 bp in length. PCR was carried out using Index (X) Primer, Universal PCR primers, and Phusion High-Fidelity DNA polymerase. Finally, PCR products were purified using an AMPure XP system, and the Agilent Bioanalyzer 2100 system evaluated the quality of the library. According to the manufacturer’s instructions, the TruSeq PE Cluster Kit v3-cBot-HS (Illumia) was used to cluster the index-coded sample data on a cBot Cluster Generation System. The library preparations were sequenced on an Illumina Hiseq platform after cluster creation, and paired-end reads were produced.

### Quality control and gene annotation

Fastq format raw data (raw reads) were initially processed using internal Perl scripts. Clean data (clean reads) were obtained in this stage by eliminating adapter-, ploy-, and low-quality readings from the raw data. The clean data’s Q20, Q30, GC-content, and sequence duplication levels were all estimated at the same time. The basis for each downstream analysis was clean and high-quality data. Trinity was used to assemble the transcriptome based on the left.fq and right.fq (Grabherr et al., 2011), with the default values of min_kmer_cov and all other parameters. The quantitative assessment of assembly and annotation completeness were performed using Benchmarking Universal Single-Copy Orthologs (BUSCO) (Simão et al., 2015).

The obtained genes were mapped using public databases, including Gene Ontology (GO), KEGG, Clusters of Orthologous Groups (COG), Non-redundancy Nucleic Acid (Nr), Nucleotide Sequence (Nt), Swiss-Prot, and Protein Family (Pfam). The animal TF database (animalTFDB 2.0) was used to predict transcription factors (TFs) for the unigenes obtained from sequencing. Gene expression levels were calculated using the RSEM (RNA-Seq by Expectation Maximization) software program (Li and Dewey, 2011).

### Identification and functional enrichment of differentially expressed genes (DEGs)

The DESeq R package was utilized to analyze the differential expression between two groups. The Benjamini and Hochberg method for reducing the false discovery rate was used to modify the resulting *p* values. DESeq identified genes classified as differentially expressed (DEGs) when their adjusted *p*-value < 0.05. The innate immune response-related genes (such as AMPs, PRRs, and signaling pathways) were screened according to the database’s annotation information, and the fragments per kilobase of transcript per million mapped reads (FPKM) values were used to define the levels of their gene expression. The generated collection of DEGs was clustered using the K-means clustering analysis (a traditional clustering algorithm frequently employed in large-scale clustering data) using the log2(ratios) of the genes’ relative expression levels (Howe et al., 2011). The GOseq R packages based on Wallenius non-central hyper-geometric distribution were used to implement the GO enrichment analysis of the DEGs, which can correct gene length bias in DEGs. We employed the KOBAS software to examine the statistical enrichment of DEGs in KEGG pathways (Xie et al., 2011). The relative abundance of the identified KO (KEGG ontology) modules that were created based on the canonical KEGG pathway maps was performed using heatmap.

### Protein-protein interaction network

To further uncover the putative mechanism following *E. coli* treatment, we constructed a protein-protein interaction (PPI) network based on the identified DEGs. The String database (https://string-db.org/) was utilized to pinpoint interactive nodes and edges. The top ten hub genes examined by Cytoscape cytoHubba were displayed in the PPI network using the Maximum Clique Centrality (MCC) method. The entire network was built using PPI data with a string score > 700. Cytoscape was used to visualize the generated network (https://cytoscape.org/).

### Quantitative real-time PCR validation

We carried out a qRT-PCR verification of randomly chosen genes in tick samples to validate the accuracy of RNA-seq results. Reverse transcription was performed using a High-Capacity cDNA Reverse Transcription Kit (Thermo Fisher Scientific Inc., USA) with a total of 1 µg total RNA from each sample. The qRT-PCR experiment was done using a combination of cDNA, forward and reverse sequence-specific primers, and iTaqTM Universal SYBR®Green Supermix (Biorad Laboratories, Hercules, CA, USA). A 95°C cycle lasted for three minutes, followed by 45 cycles of 95°C (10 s), 40 cycles of 60°C (60 s), and 35 to 40 cycles of 65 to 95°C (0.5°C increments at 2 to 5 sec/step). The reference gene was a V-ATPase subunit C (*VAC*) gene (Maldonado-Ruiz et al., 2021). The foldchange of identified genes was calculated using the relative quantitative approach (2-^△△CT^), and the primers are reported in **Table S1**.

### Data analysis

The read counts and FPKM values of each gene were calculated (Li and Dewey, 2011), respectively. The normality of the data was assessed using the Shapiro-Wilk or Kolmogorov-Smirnov test, while the Brown-Forsythe test confirmed the homogeneity of variance. With these assumptions met, a one-way analysis of variance (ANOVA) was conducted to determine significant differences among group means. The “pheatmap” and “ggplot2” R packages were used to show the DEG level. For figure processing and grouping, Adobe Photoshop CS6 and Adobe Illustrator CC (https://www.adobe.com/) were utilized.

## Results

### cDNA library construction, assembly, and annotation

**Figure 1:**
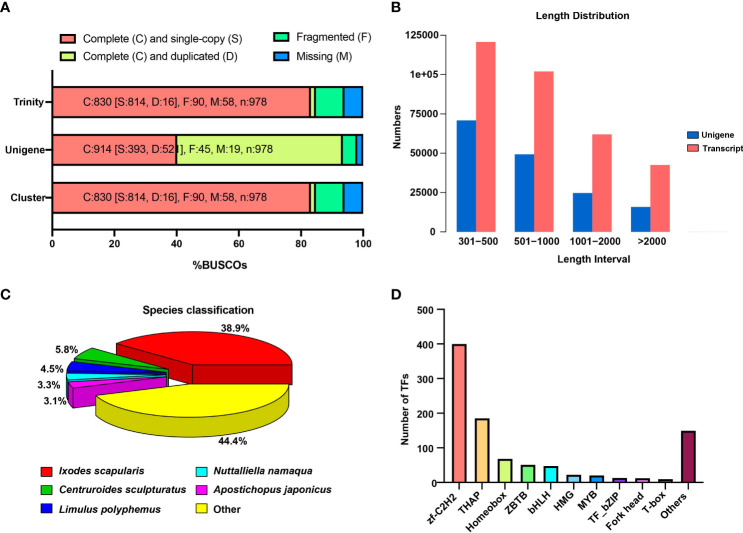
Identification of DEGs and general RNA-seq data patterns. **(A)** The Benchmarking Universal Single-Copy Orthologs (BUSCO) analysis shows the transcriptome assembly’s completeness. **(B)** The distribution of the number and length of transcripts and unigenes produced from the RNA-seq data. **(C)** Similar species of best Hit against Nr database. **(D)** The number of identified transcription factors (TFs) in the tick transcriptome.

The Trinity assemblies and unigenes were subjected to the BUSCO analysis (**Figure 1A**). Out of 982 evaluated BUSCOs for the clusters, 814 (83.2%) were complete (single-copy), demonstrating the completeness and quality of the Trinity assemblies and unigenes. The majority of transcripts (120,686) and unigenes (70,853) were within the 300–500 base pair (bp) range (**Figure 1B**). The findings revealed that *Ixodes scapularis*, a well-known black-legged tick, was one of the most similar species to *A. americanum*, followed by *Centruroides sculpturatus*, *C. sculpturatus*, and *Limulus polyphemus* (**Figure 1C**). The majority of unigenes were engaged in cellular processes, metabolic processes, cellular anatomical entities, and binding, according to GO functional enrichment (**Figure S2**). A total of 1,257 unigenes were identified using KEGG pathway analysis as being involved in signal transduction, 709 in the endocrine system, and 481 in the immune system (**Figure S3**). Similar to KEGG, KOG’s functional classification revealed that posttranslational modification, protein turnover, and chaperones were the next most enriched functional annotations, and with signal transduction mechanism the most enriched function overall (**Figure S4**). We further classified the transcriptional factors into 61 categories, and 400 unigenes were annotated as zinc finger Cys2-His2 (zf-C2H2) and 186 as thanatos-associated protein (THAP) (**Figure 1D**).

### Global quantification analysis and DEGs identification

**Figure 2:**
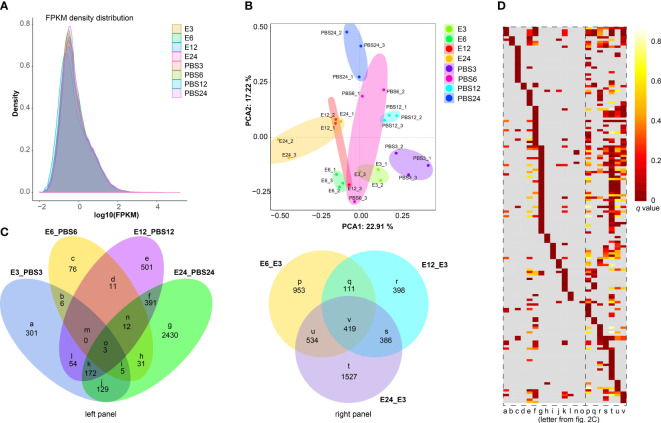
Global quantification analysis and DEGs identification. **(A)** FPKM density distribution comparing the expression levels of PBS-treated and *E coli-treated* samples, illustrating the distribution of gene expression values. **(B)** A principal component analysis (PCA) graphic shows how distinct cDNA libraries differ. **(C)** Venn diagrams with the control group identified in each panel show the number of overlapping DEGs in various comparisons. **(D)** Heatmaps exhibiting significantly altered Gene Ontology (GO) terms in various subsets of DEGs obtained from the Venn diagrams **(C)**, with the logarithm of the false discovery rate (log (FDR)) values indicating the significance of GO term enrichment. Detailed GO annotations are provided for these terms in **Table S2**.

The comparison of gene expression profiles is shown by the FPKM density value (**Figure 2A**). As shown in **Figure 2B**, samples from the *E. coli*-treated groups significantly differed from the control group, indicating that the differences between the groups were evident. In total, 670 (580 up-regulated and 90 down-regulated), 144 (67 up-regulated and 77 down-regulated), 1,144 (772 up-regulated and 372 down-regulated), and 3,173 (2,048 up-regulated and 1,125 down-regulated) DEGs were identified from the comparisons of E3 vs PBS3, E6 vs PBS6, E12 vs PBS12, and E24 vs PBS24, respectively (**Figure S5**, **Tables S3**-**S6**). The top 10 altered genes in each comparison included the genes for defensin 2, ATP-dependent RNA helicase DDX4, and placenta growth factor (**Figure S6**).

Pair-wise comparisons were then used to compare the overlapped DEGs. For instance, in “E3 vs PBS3/E6 vs PBS6” (**Figure 2C**, subset b) and “E12 vs PBS12/E24 vs PBS24” (**Figure 2C**, subset f), 6 DEGs and 391 DEGs, respectively, were overlapped. The total number of DEGs that were solely expressed in the “E3 vs PBS3” (**Figure 2C**, subset a), “E6 vs PBS6” (**Figure 2C**, subset c), “E12 vs PBS12” (**Figure 2C**, subset e), and “E24 vs PBS24” (**Figure 2C**, subset g) groups was 301, 76, 501, and 2,430, respectively. Among the *E. coli*-treated groups, a total of 953, 398, and 1,527 DEGs were identified exclusively in the comparisons of “E6 vs E3”, “E12 vs E3”, and “E24 vs E3”, respectively (**Figure 2C**, subset p, r, t). The gene clustering divergence of the overlapped DEGs in various subgroups was then investigated using GO enrichment analysis (**Figure 2D**, **Table S2**). The results revealed that subsets a-o exhibited a variety of functional patterns. Subset a, for instance, displayed patterns related to “cytokine activity” and “acid phosphatase activity.” Subset g was enriched in “lipid transport”, “suppression by virus of host transcription”, “toxin biosynthetic process”, etc. Subsets p-v showed similar functional patterns, including “lysozyme activity”, “glycerol metabolic process”, and “bacterial-type flagellum assembly”. We had speculated that *E. coli* treatment would alter the gene expression and functional patterns in ticks compared with the control (**Figure 2C**, left panel), and the altered functions displayed conserved patterns to some extent within 24 h of treatment (**Figure 2C**, right panel).

### K-means clustering analysis

**Figure 3:**
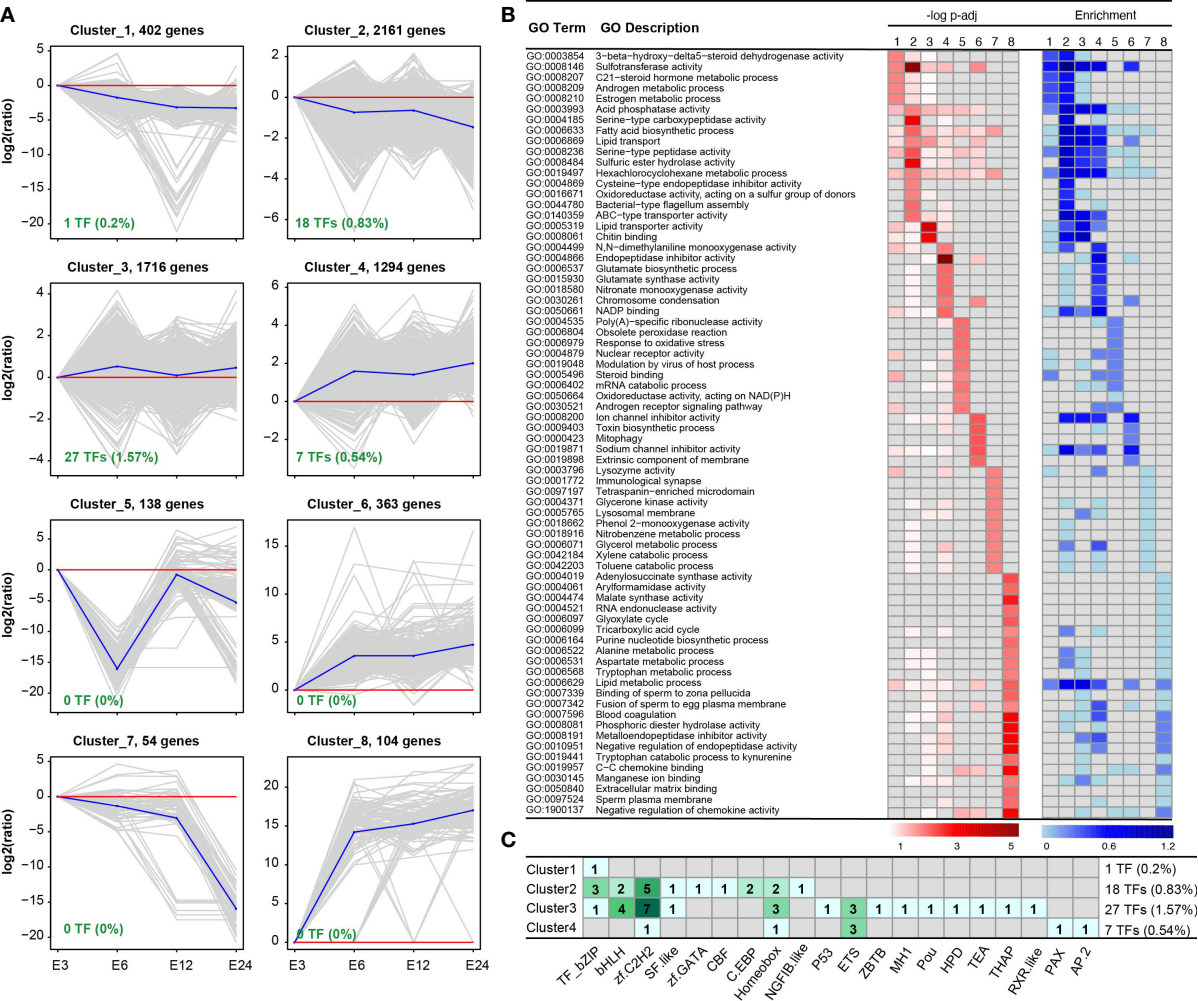
K-means clustering analysis of DEGs obtained from the four time frame treated groups. **(A)** K-means clustering analysis identified eight distinct clusters based on the expression patterns of DEGs. **(B)** Functional annotation of the DEGs within each cluster provides insights into the biological processes and molecular functions associated with the gene expression patterns. **(C)** The distribution and count of transcriptional factors (TFs) within the eight clusters, highlighting the presence or absence of TFs in each cluster (clusters 5-8 did not contain TFs).

DEGs were grouped using K-means analysis to find the expression pattern of the tick’s response to *E. coli* treatment within 24 h. Among eight clusters, three clusters (1, 2, and 7) represented down-regulation, and three clusters (4, 6, and 8) showed up-regulation in the 6 h, 12 h, and 24 h groups compared with the 3 h group (**Figure 3A**). While cluster 5 reversed the expression patterns, cluster 3 showed up-regulation patterns in DEGs in the 6h and 24 h time frames. “Sulfotransferase activity,” “acid phosphatase activity,” and “lysozyme activity” were annotated subsets 1, 2, and 7 according to GO enrichment analysis (**Figure 3B**). Based on the K-means clustering analysis, additional GO biological processes were discovered, including “glutamate synthase activity” and “glutamate biosynthetic process” in cluster 4, “response to oxidative stress” in cluster 5, and “mitophagy” in cluster 6. The major TF subfamilies observed in each cluster were also studied, and they were highly sensitive to clusters 1-4, especially TF-bZIP, bHLH, zf-C2H2, homeobox, and ETS (**Figure 3C**).

### Genes expression profiles involved in innate immune response

**Figure 4:**
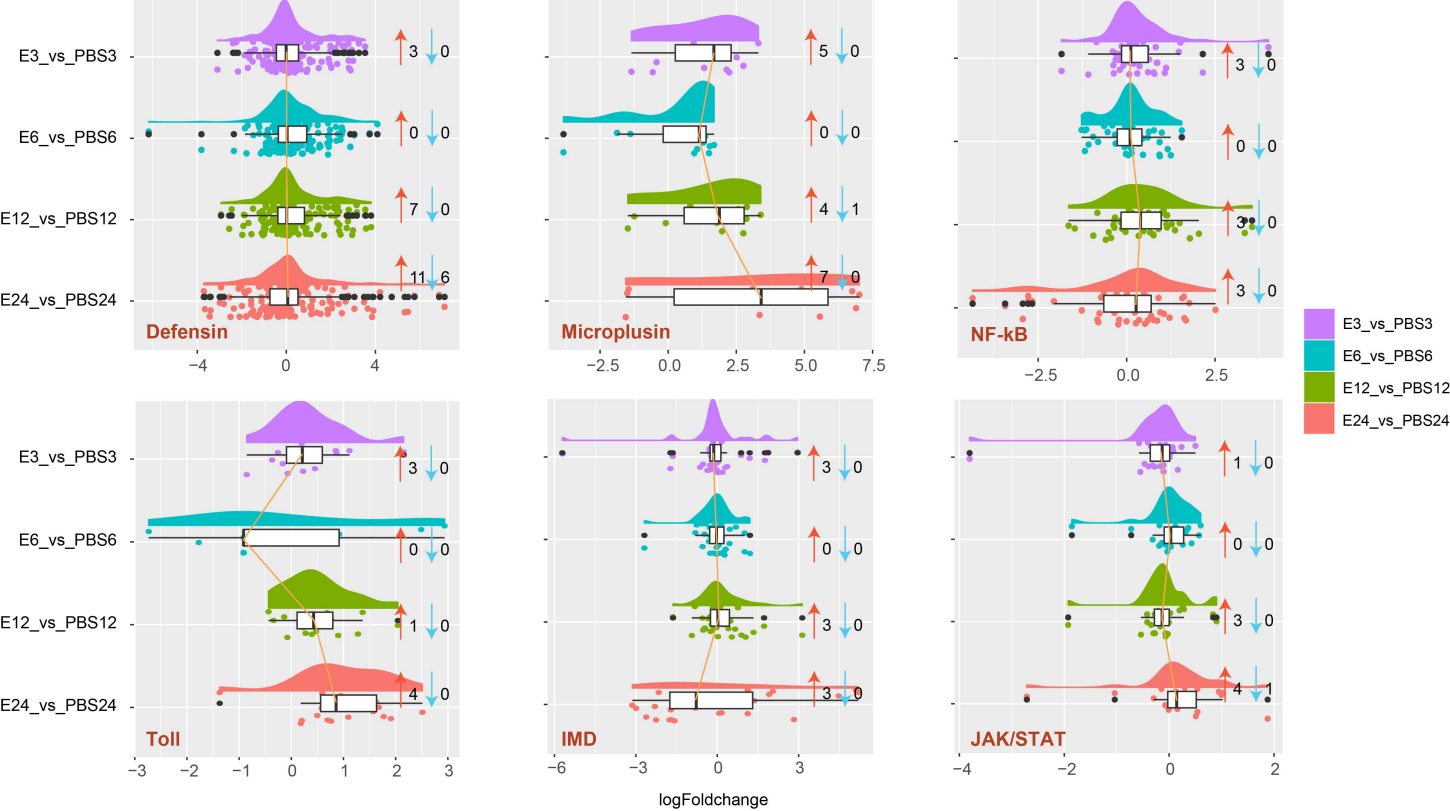
Raincloud plot analysis of genes involved in the innate immune response in *A. americanum*. The plot depicts the expression patterns of genes belonging to the defensin, microplusin, Nf-kB, toll, IMD, and JAK/STAT families. The data density and distribution are represented using a half-violin graph (cloud), while individual data points are depicted beneath the cloud (rain). Thunder (lines connecting median values for many categories) and an umbrella (boxplot) provide further summary statistics. The number of up-regulated genes is indicated by the red arrow, while the number of down-regulated genes is indicated by the blue arrow.

According to the results, *A. americanum*’s distinct gene functions were affected by *E. coli* exposure. As a result, we investigated the effects of *E. coli* treatment on the innate immune systems, including AMPs (such as defensin and microplusin), NF-kB, Toll, IMD, and JAK/STAT pathways (**Figure 4**). For each gene family, there were distinct global expression tendencies. It is interesting to note that defensin, microplusin, NF-kb, Toll, IMD, and JAK/STAT were associated with a total of 3, 5, 3, 3, 3, and 1 up-regulated genes at the early stage (3 h). In the E6 vs. PBS6 treatment, no DEGs were found. However, compared to the 3 h treatment, the 12 h and 24 h treatments revealed comparable DEG patterns, and more DEGs involved with defensin, microplusin, Toll, and JAK/STAT found in the 24 h group.

### KEGG pathway analyses based on the DEGs

**Figure 5:**
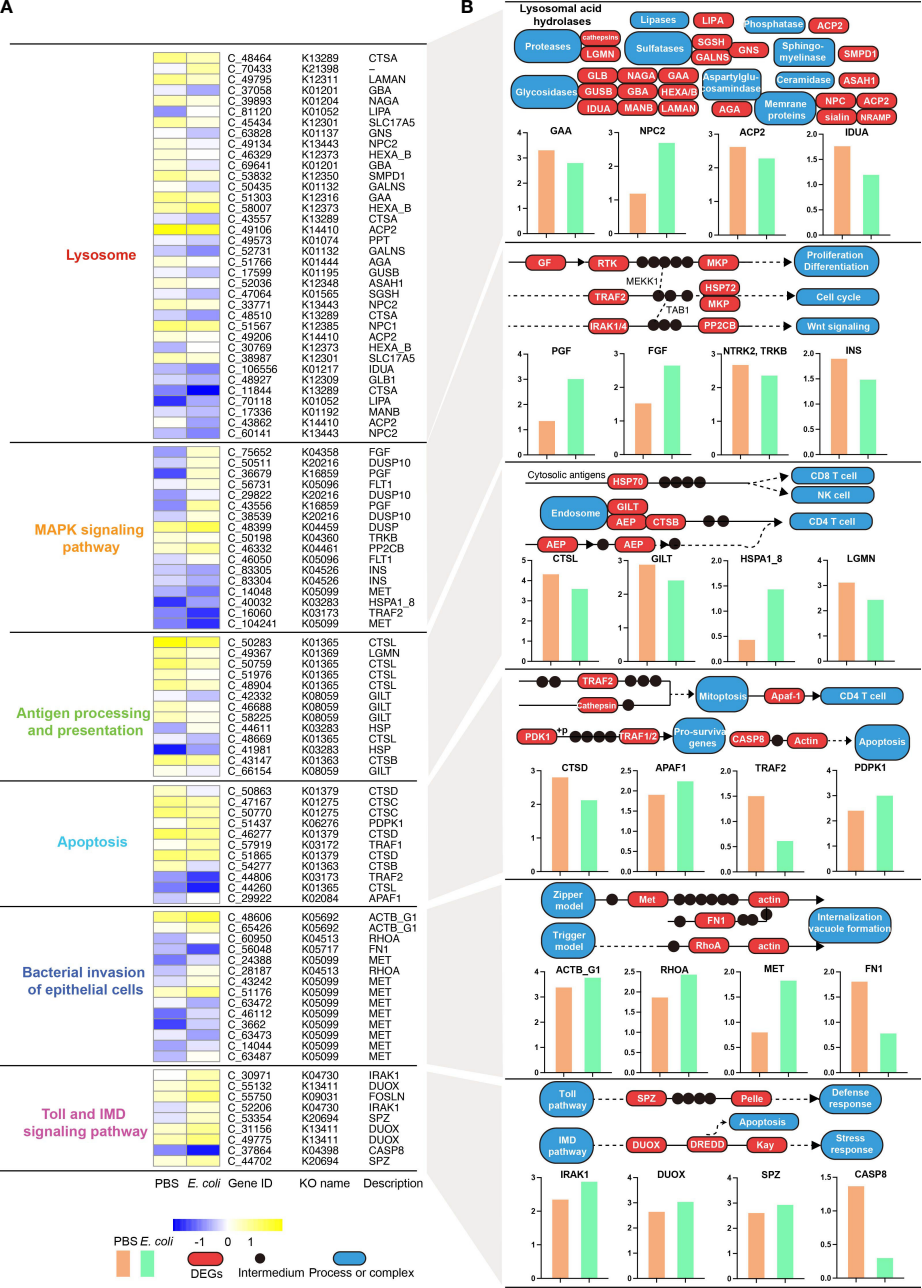
Integrative analysis of differentially expressed genes (DEGs) using KO genes and KEGG modules at 24 h of ***E*.** *coli* challenge. **(A)** The relative expression levels of KO genes are associated with six pathways, including the lysosome, MAPK signaling pathway, antigen processing and presentation, apoptosis, bacterial invasion of epithelial cells, Toll and IMD signaling pathways. **(B)** Modules represent representative KO genes based on the classical KEGG pathway maps. The red box indicates an altered KO module (DEG), the dot represents an intermediate, and the blue rounded rectangle represents an indirectly altered biological process. The bar charts show the relative abundance of each KO module using log(readcount) values. .

The results showed that *A. americanum* responded considerably to *E. coli* treatment *via* similar functional pathways at different time points and that treatment may influence the number of enriched DEGs. Therefore, further investigated the KEGG pathways that were DEG-enriched in the early (3 h) and late (24 h) stages of *E. coli* challenge. The E6 vs PBS6 and E12 vs PBS12 comparisons did not result in any changes to the KEGG pathways (**Figure S7**). *A. americanum* dramatically increased the expression levels of genes involved in the “MAPK signaling pathway”, “bacterial invasion of epithelial cells”, and “apoptosis” after 3 h of *E. coli* exposure. Numerous up-regulated genes, such as *SHC1* (SHC-transforming protein 1), *PROX1* (prospero homeobox 1), *FN1* (fibronectin 1), *MET* (proto-oncogene tyrosine-protein kinase Met), *FOSLN* (fos-like antigen, invertebrate), *DUSP10* (dual specificity phosphatase 10), and others, were responsible for the regulation of the pathways (**Figure S8**). Intriguingly, the E24 vs PBS24 analysis also revealed that the three KEGG pathways were significantly altered following *E. coli* treatment. DEGs were additionally engaged in the lysosome, antigen processing and presentation, and in the Toll and IMD signaling pathways in the E24 vs PBS24 comparison (**Figure 5**). The findings specifically demonstrated that 26 DEGs implicated in a number of enzyme functions, including lysosomal alpha-glucosidase (*GAA*), lysosomal acid phosphatase (*ACP2*), and Niemann-Pick C2 protein (*NPC2*), were significantly changed, compromising lysosome activity. The MAPK pathway was affected by the alteration of seventeen DEGs, including insulin (*INS*), neurotrophic tyrosine kinase receptor type 2 (*TRKB*), fibroblast growth factor (*FGF*), placenta growth factor (*PGF*), and fibroblast growth factor (11 up-regulated and 6 down-regulated). A total of 13 DEGs in all, including those encoding numerous important enzymes like cathepsin L (*CSTL*), interferon gamma-inducible protein 30 (*GLIT*), legumain (*LGMN*), etc., were distinctively changed and affect antigen processing and presentation. Apoptosis was obviously affected at 24 h of treatment, as evidenced by the up-regulated genes for cathepsin D (*CTSD*), apoptotic protease-activating factor (*APAF1*), TNF receptor-associated factor 1 (*TRAF2*), and 3-phosphoinositide dependent protein kinase-1 (*PDPK1*). Two canonical invasive models (the zipper and the trigger) showed up-regulated patterns to affect internalization and vacuole formation, and 14 DEGs (11 up-regulated and 3 down-regulated) were shown to be involved in the regulation of bacterial invasion of epithelial cells. Eight up-regulated genes (e.g., interleukin-1 receptor-associated kinase 1 (*IRAK1*), dual oxidase (*DUOX*), and protein spaetzle (*SPZ*)) and one down-regulated gene (caspase 8 (*CASP8*)) that affect the defensive response and apoptosis were found to be enriched in the Toll and IMD signaling pathways.

### PPI modulatory network among the DEGs

**Figure 6:**
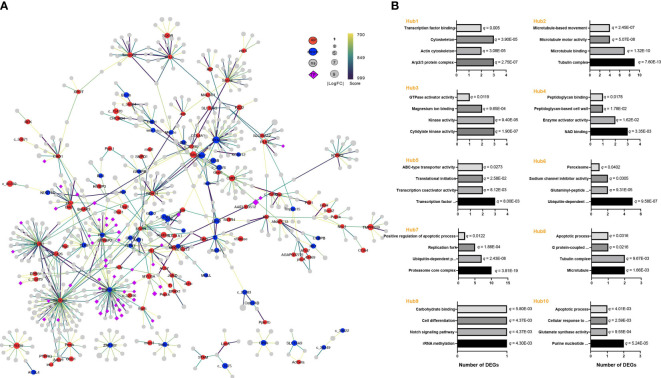
Response of the protein interaction network in *A americanum* at 24 h of *E*. *coli* challenge. **(A)** The network of protein interactions and modular analysis. The diameters of the nodes reveal the relative amounts of gene expression. The edges depict the patterns of associations between particular genes. The node color (red for up-regulated, blue for down-regulated, and gray for non-significant genes) shows the significance of gene expression changes. Transcription factors are shown by purple diamond nodes. **(B)** GO functional annotation of the nodes obtained from the hub gene analysis.

The effects of *E. coli* on *A. americanum* were next examined by looking at protein interaction networks. Compared to the E3 vs. PSB3 comparison, which had 153 nodes and 149 connections (**Figure S8**), the created network for the E24 vs. PBS24 comparison had 708 nodes and 803 interactions (**Figure 6A**). Also shown are the top ten hub genes and a GO enrichment analysis for the modules that these genes controlled (**Figure S9**, **Figure 6B**). Interestingly, hub 1, 2, and 8 regulated the functions of “cytoskeleton” (3 DEGs), “actin cytoskeleton” (2 DEGs), “tubulin complex” (11 DEGs), “microtubule binding” (7 DEGs), and “microtubule” (2 DEGa) (*q* < 0.05). The regulation of the apoptotic processes, including “positive regulation of apoptotic process” (2 DEGs) and “apoptotic process” (2 DEGs) (*q* < 0.05), was distinctively associated with hubs 7, 8, and 10. The DEGs implicated in “kinase activity” (3 DEGs) and “GTPase activator activity” (1 DEG) were abundant in hub 3 (*q* < 0.05).

### qRT-PCR validation

Four genes were chosen for qRT-PCR quantification to further confirm the protein encoding genes in the transcriptome. The results illustrated reliability of the RNA-seq data by demonstrating that the expression profiles of the selected genes were consistent with foldchange values computed in the transcriptome analysis (**Figure S10**).

## Discussion

The objective of the present research was to examine the molecular and immune system responses of *A. americanum* following treatment with *E. coli* by thorough transcriptome analysis through 24 hours. Within 3 h, 6 h, 12 h, and 24 h after *E. coli* treatments, a large number of DEGs were found in *A. americanum*, demonstrating the integrative responses of ticks to bacterial challenge in a time-dependent manner. Our investigation of gene expression using FPKM density values led to the discovery of different gene expression profiles between the groups treated with *E. coli* and the control group. Significant changes in gene expression between the *E. coli*-treated groups are indicated by the separation of samples and their close grouping within each treatment time point. These results align with earlier research that showed significant transcriptional alterations in ticks in response to bacterial challenges (Xu et al., 2016; Kim et al., 2018). In BME26 cells, a cell lineage derived from the embryos of the cattle tick *Rhipicephalus microplus*, the challenge with the gram-negative bacterium *Enterobacter cloacae* induced the expression of the majority of the analyzed genes after 6 h, and most immune-related genes (i.e., Toll, IMD, and JAK/STAT) continued to be induced until 72 h (Rosa et al., 2016), indicating that there is a conserved immune response mechanism present in both the cell line and the whole tick. Similarly, *E. coli* treatment induced the aggregation of bacteria with cellular material and triggered the immune system in the tick *Dermacentor variabilis* (Acari: Ixodidae). Hence, while some *E. coli* strains do not represent a natural tick-borne pathogen, they are valuable for investigating fundamental aspects of tick immunity. It provides a simplified and controlled system to explore general immune mechanisms and pathways without the complexities associated with studying diverse and specialized tick-borne pathogens.

Based on the pair-wise analysis, our results revealed that subsets p-v shared comparable functional patterns, particularly for lysozyme activity. The development of fecundity and immunity in the tick species *Haemaphysalis longicornis* and *Ornithodoros moubata* have been linked to the antimicrobial enzyme lysozyme’s critical role in dissolving bacterial cell walls (Grunclová et al., 2003; Koloski and Cassone, 2022). This defense against bacterial pathogens is facilitated by lysozyme, which has been suggested to have an impact on these ticks’ development. The change in lysozyme activity after exposure to *E. coli* is an important discovery since it raises the possibility of a potential mechanism by which ticks react to bacterial challenges. This finding is consistent with previous research that has implicated lysozyme activity in the immune response against bacterial challenges from pathogens and non-pathogens, such as *E. coli* (Simser et al., 2004; Hajdušek et al., 2013; Kao et al., 2016). It is intriguing that the KEGG analysis confirmed that 26 DEGs were altered, potentially influencing the activities of lysosome encoding genes such as *GAA*, *ACP2*, and *NPC2*. Since ticks may upregulate their lysozyme production or release in response to bacterial challenge, the observed shift in lysozyme activity is suggested to be a putative innate immune strategy for *A. americanum* to counteract the *E. coli* challenge.

Eight unique clusters were identified by the clustering analysis; the down-regulated genes were represented by clusters 1, 2, and 7, while the up-regulated genes were represented by clusters 4, 6, and 8 in the 6-hour, 12-hour, and 24-hour treatment groups as compared to the 3-hour group. Cluster 6, showing an increased expression pattern following the time treatment, was enriched with genes involved in “mitophagy,” a cellular procedure involved in the elimination of damaged mitochondria (Gustafsson and Dorn, 2019). According to this discovery, ticks may initiate mitophagy in response to *E. coli* treatment, maybe as a defense mechanism to remove damaged mitochondria and preserve cellular homeostasis. Other studies have also noted the role of mitophagy in the tick’s response to bacterial challenge, highlighting the significance of this mechanism as a conserved method of immunological modulation and cellular quality control (Chang et al., 2022; Verbeke et al., 2022). Additionally, compared to the 6 h and 24 h treatments, the expression levels of “response to oxidative stress” genes were higher in the 3 h and 12 h treatments. The activation of antioxidant defense mechanisms in response to the reactive oxygen species produced during the initial stages of bacterial challenge may be part of this response. In addition, many TFs are key regulators of cellular metabolism, proliferation, and stress resistance. Some TFs, such as Forkhead, zf-C2H2, and ZBTB, are able to regulate innate immune mechanisms in epithelial cells and hosts upon pathogen invasion (Seiler et al., 2013; Yin et al., 2022). The finding that the major TF subfamilies, including TF-bZIP, bHLH, zf-C2H2, homeobox, and ETS, were highly specific to clusters 1-4 suggests their important regulatory roles in the gene expression changes observed in those clusters. These TF subfamilies are known to be involved in various biological processes and can influence the expression of multiple genes. Their clustering specificity indicates their potential involvement in mediating the transcriptional responses of ticks to *E. coli* treatment, potentially playing critical roles in orchestrating the observed gene expression patterns and functional changes.

Understanding the tick’s capacity to defense against bacterial diseases is made possible by the changes in the innate immune system that have been documented in response to *E. coli* treatment in *A. americanum*. Genes linked to AMPs, like defensin and microplusin, were upregulated as a significant feature of this tick’s immune response. Small proteins called AMPs are critical components of the innate immune defense of organisms in all species (Andreu and Rivas, 1998; Bulet and Stocklin, 2005; Falanga et al., 2016). These peptides have a remarkable capacity to locate and kill bacteria and other microbial challenges directly. The up-regulation of AMP-related genes showed that *A. americanum* develops an effective antimicrobial response in response to *E. coli* exposure, potentially assisting in the eradication of the pathogen that is causing the challenge. The midgut and salivary glands of infected *A. aureolatum* were both found to have considerably higher levels of microplusin expression (Martins et al., 2019). Similarly, treatment with *E. coli* resulted in changes to the baseline expression levels of pattern recognition receptors, opsonins, lysozymes, AMP genes, and immune response genes in the larvae of *Drosophila* (Marcu et al., 2011). Our research discovered that, in response to *E. coli* treatment, different genes involved in important signaling pathways, such as NF-kB, Toll, IMD, and JAK/STAT, expressed themselves differently. It is well recognized that these signaling pathways play a crucial role in controlling immune responses and coordinating the expression of genes relevant to immunity (Kopáček et al., 2010; Fogaça et al., 2021). Additionally, under *E. coli* challenge, DUOX, a gene known for the creation of reactive oxygen species as a defensive mechanism against infectious bacteria, was upregulated, which may be a sign of a defense mechanism that has successfully reduced pathogen invasion (Ha et al., 2009).

*A. americanum* significantly up-regulated multiple KEGG pathways following the first three hours of the *E. coli* treatment, including genes involved in the bacterial invasion of epithelial cells, MAPK signaling, and apoptosis. The up-regulation of these pathways implies that defensive mechanisms against the invasive pathogen have been activated. It is interesting to note that these three KEGG pathways (MAPK signaling, bacterial invasion of epithelial cells, and apoptosis) were also shown to have undergone substantial changes in the E24 vs PBS24 comparison, demonstrating that they have remained important in *A. americanum*’s response to *E. coli* throughout time. Additional KEGG pathways impacted by *E. coli* treatment were discovered by comparing E24 to PBS24. These included pathways involved with lysosome, Toll and IMD signaling, and the processing and presentation of antigens. The findings of changes in the KEGG pathway related to “bacterial invasion of epithelial cells” and the identification of hub genes associated with cytoskeleton organization, actin, tubulin complex, and rearrangement suggest that the cytoskeleton rearrangement is a potential target in the discussion. During bacterial invasion, pathogens and host cells engage in interactions that facilitate the internalization of bacteria into host cells. The involvement of cytoskeleton-related mechanisms implies that the manipulation of cytoskeletal components could potentially influence the bacterial invasion process (Dramsi and Cossart, 1998; Sonenshine and Macaluso, 2017; Oliva Chávez et al., 2021). Kinases play a crucial role in signal transduction pathways that regulate various cellular processes, including immune responses and cytoskeleton dynamics. Alterations in kinase activity may influence the host’s ability to respond to bacterial invasion and modulate immune signaling pathways. The strictly controlled process of apoptosis, also known as programmed cell death, is essential for preserving tissue homeostasis, eliminating diseased or infected cells, and controlling immunological responses (Mansfield et al., 2017; Wang and Cull, 2022). The hub genes linked to the positive regulation of apoptotic processes also suggested that *A. americanum* apoptosis may be induced or enhanced by treatment with *E. coli*. Pathogens are taken up internally and packed into the apoptosome, causing a more effective fusion of the phagosome and lysosome, which is then followed by digestion and destruction (Willingham et al., 2007; Cervantes et al., 2008; Wang and Cull, 2022). Upregulating nucleases and enzymes in response to apoptosis activation can also cause cell death and aid in the removal of pathogens in ticks.

Our results agree with findings from earlier studies on other animals, suggesting that the observed changes in lysozyme activity, mitophagy, the MAPK signaling pathway, AMPs, apoptosis, and immune signaling pathways are possible strategies of defense against bacterial challenge. These results show how these molecular pathways are consistent across other species and advance our understanding of *A. americanum’s* immune-mediated defense mechanisms. However, it is crucial to distinguish between the effects of *E. coli* as a bacterial challenge and the effects of a natural tick-borne infection. Tick-borne pathogens are specifically adapted to infect and persist within ticks, often employing strategies to evade or modulate the tick’s immune responses. In contrast, *E. coli* is not a natural tick-borne pathogen, and the responses observed in tick-*E. coli* interactions may not fully mirror the complexity and dynamics of a natural tick-borne infection. Nonetheless, the use of *E. coli* as a model organism could provide insights into the tick’s general immune response to bacterial challenges to some extent. In order to comprehend the persistence of altered gene expression and functional pathways over lengthy stretches of time, it would be beneficial to explore the long-term consequences and dynamics of these reactions. Determining the functional importance of the discovered genes and pathways requires additional experimental validation, although functional annotations and pathway enrichment analyses offer insightful information. Future research may use methods like gene overexpression or knockdown to examine the precise functions of important genes and pathways in the immune response of *A. americanum*. Future research, including additional omics techniques like proteomics and metabolomics, and the study involved in gram-positive bacteria and tick-borne pathogens may provide a more thorough knowledge of the innate immune system in *A. americanum*.

## Conclusion

The current work examined changes in gene expression, functional pathways, and protein interactions in the lone star tick *A. americanum* after exposure to *E. coli*. *E. coli* challenge changed the expression of genes related to the innate immune system, KEGG pathways, and protein-protein interaction networks. Functional pathways related to apoptosis, mitophagy, MAPK signaling, lysozyme activity, antigen processing and presentation, Toll and IMD signaling, and bacterial invasion were significantly changed under *E. coli* challenge. The knowledge gathered from this study may be used in better understanding of tick innate immune system and creating fresh approaches to tick control and management.

## Data availability statement

The transcriptomic datasets used in the research are available in Sequence Read Archive (https://www.ncbi.nlm.nih.gov/genbank/) with the accession number PRJNA982785.

## Ethics statement

Ethical review and approval was not required for our study of ticks in accordance with local legislation and institutional requirements. Animal welfare of arthropods used in research and teaching are not covered by federal, state, or local agencies.

## Author contributions

QS: conceptualization, methodology, and reviewing. BL: writing original draft, data analysis, and visualization. JL: materials preparation, experimental design, investigation, and reviewing. BN: materials preparation, experimental design, investigation, and reviewing. DA and BB: supervision, editing, and reviewing. All authors contributed to the article and approved the submitted version.
