# Supplementary material for: Integrative analysis highlights molecular and immune responses of tick Amblyomma americanum to Escherichia coli challenge
Source: Front Cell Infect Microbiol. 2023 Jul 31;13:1236785. doi: 10.3389/fcimb.2023.1236785 (PMC10424933; doi:10.3389/fcimb.2023.1236785)
Supplement: Supplementary file 1 [file DataSheet_1.docx]

**Integrative analysis highlights molecular and immune responses of tick *Amblyomma americanum* to *Escherichia coli* stress**

Bo Lyu^1^, Jingjing Li^1^, Brigid Niemeyer^1^, Deborah M. Anderson^2^, Brenda Beerntsen^1,2^, Qisheng Song^1,*^

^1^Division of Plant Science and Technology, University of Missouri, Columbia, MO 65211, USA

^2^Department of Veterinary Pathobiology, University of Missouri, Columbia,

MO, United States

^*^ Corresponding author

Qisheng Song; E-mail: SongQ@missouri.edu; Phone number: +1 5738829798;


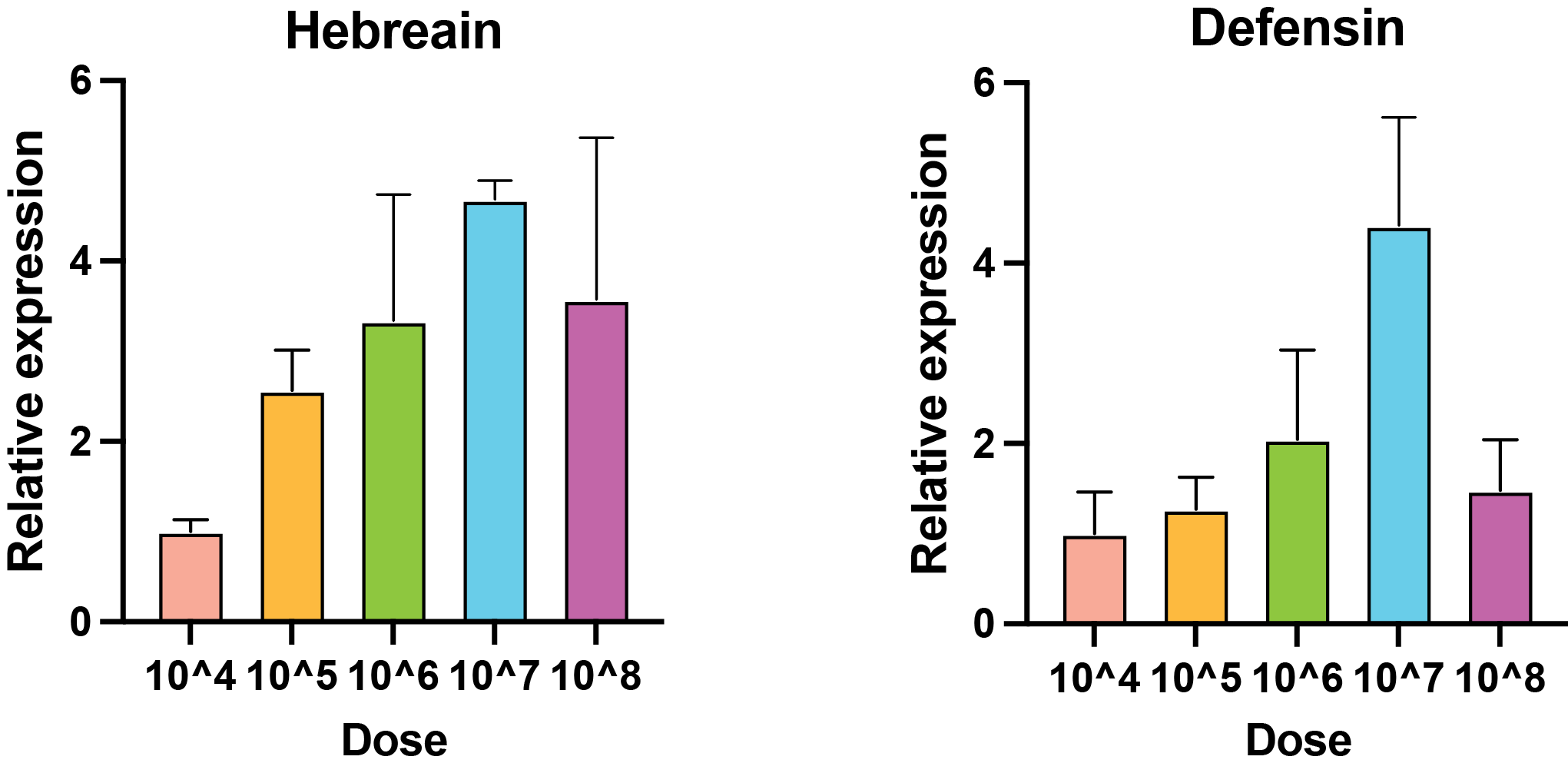


**Figure S1. qRT-PCR analysis of two antimicrobial peptide encoding genes with dose-dependent effects.**


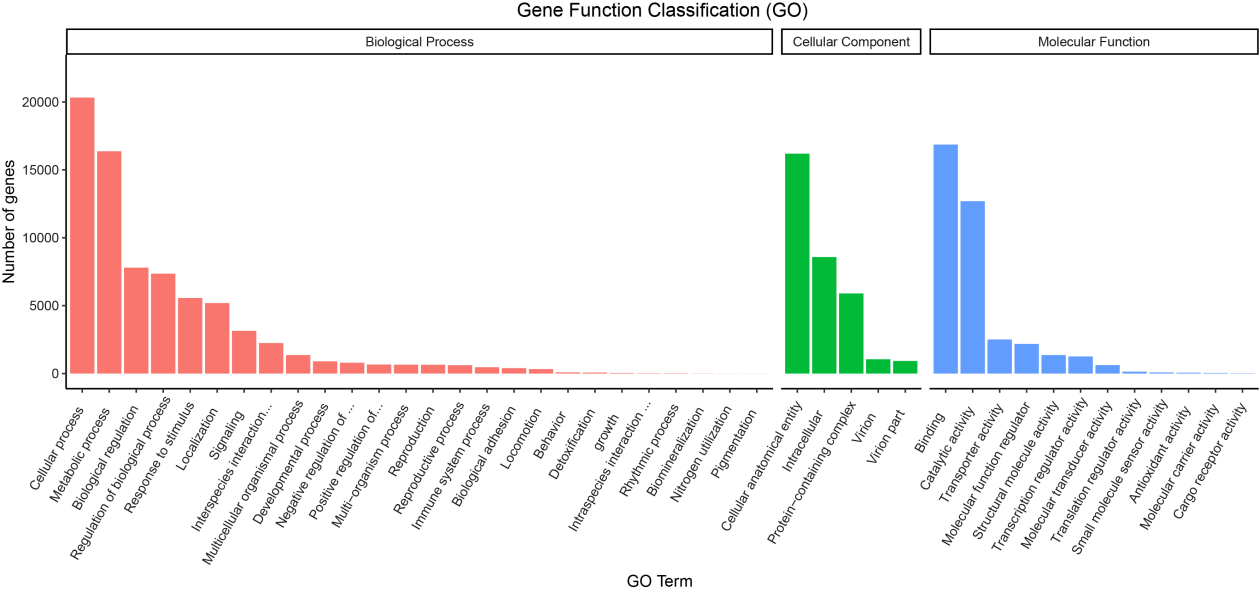


**Figure S2. Gene Ontology (GO) analysis based on bar plots showing the distribution of unigenes in different categories.** The y-axis represents the number of unigenes, while the x-axis represents the three types of GO analysis: molecular function, biological process, and cellular component. The bars depict the frequency of unigenes assigned to each specific GO category, providing insights into the functional annotation and classification of the identified genes.


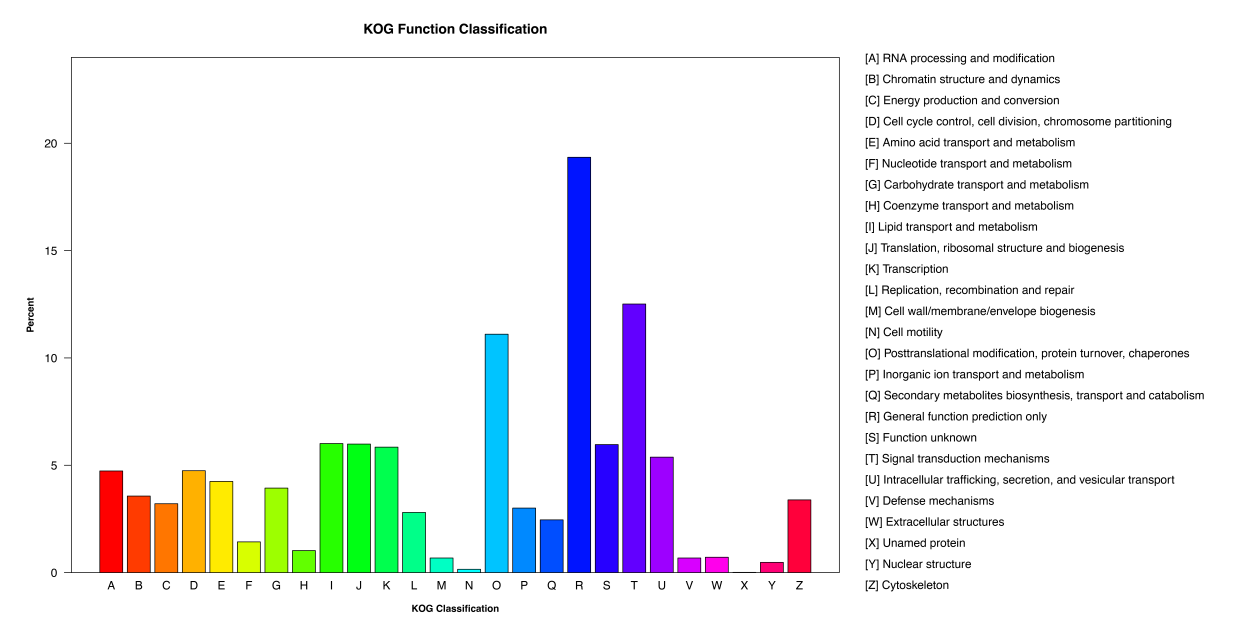


**Figure S3. Clusters of Orthologous Groups (KOG) analysis based on bar plots illustrating the distribution of unigenes in different functional categories.** The y-axis represents the percent of unigenes, while the x-axis represents the various functional categories of KOG analysis.


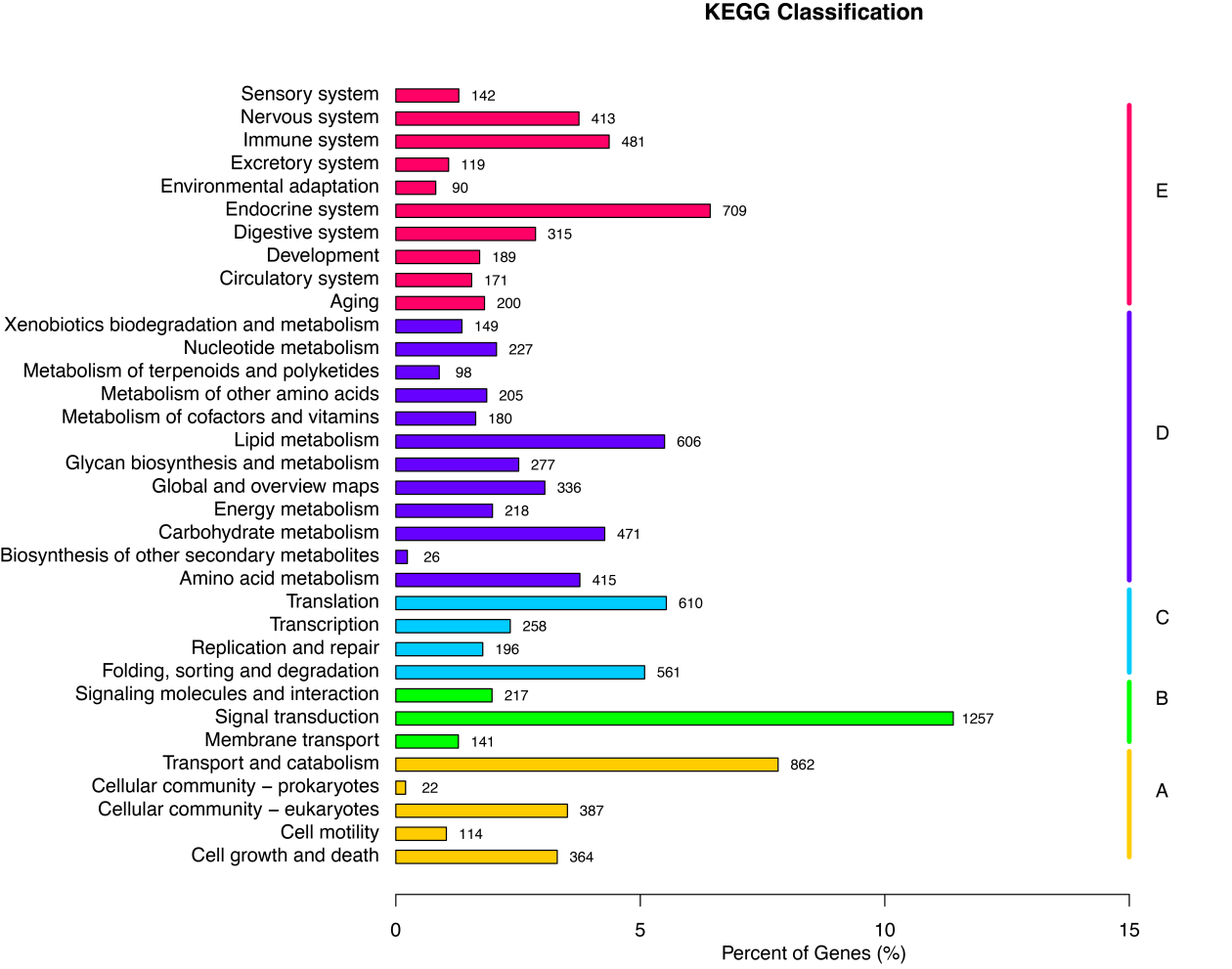


**Figure S4. KEGG analysis based on stacked bar plots illustrating the distribution of genes in different KEGG families.** The x-axis represents the percentage of genes, while the y-axis represents the five major KEGG families, including Cellular Processes, Environmental Information Processing, Genetic Information Processing, Metabolism, and Organismal Systems.


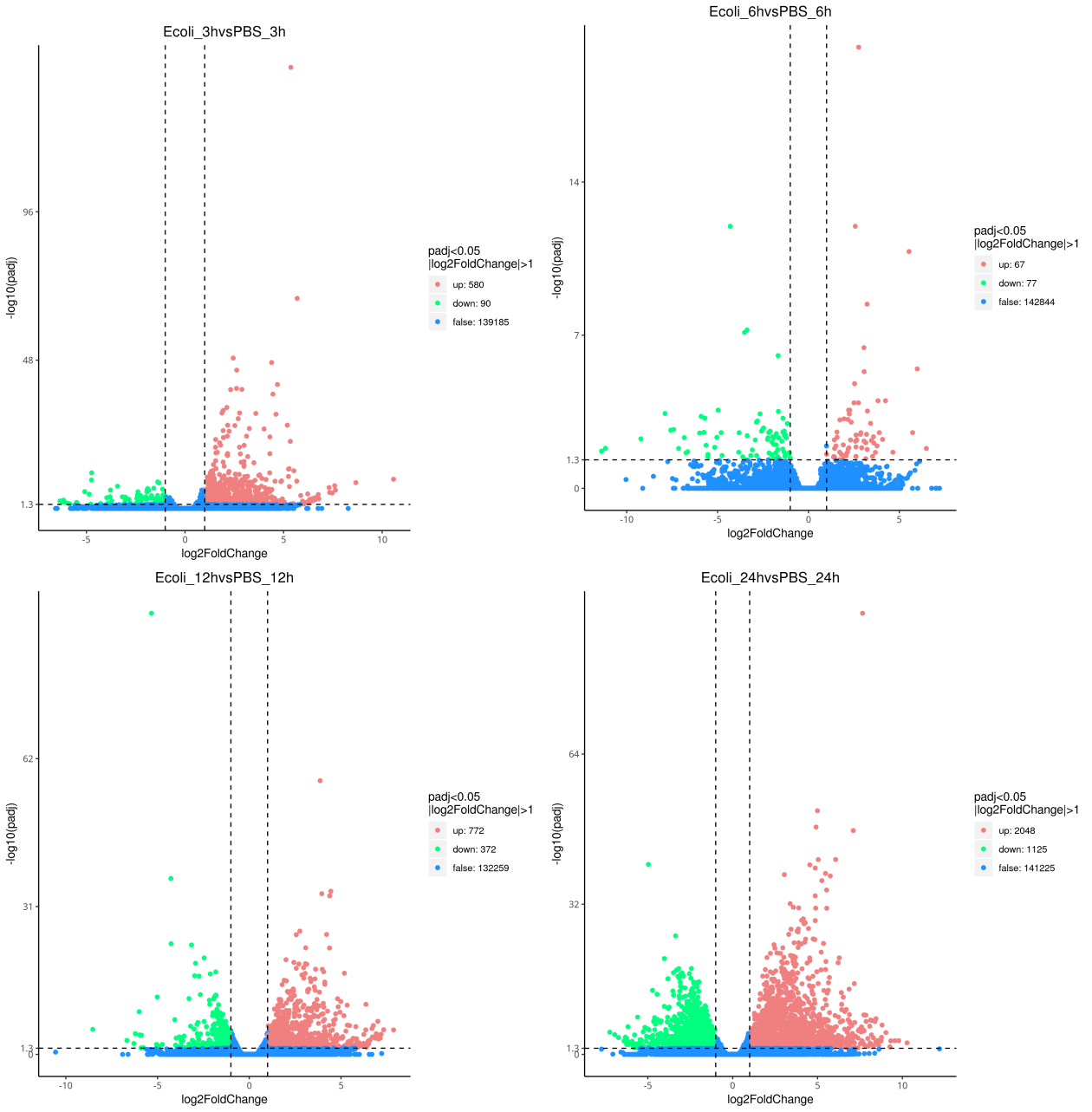


**Figure S5. Volcano plot displaying the number of DEGs in four comparisons.** The x-axis represents the log2 fold change (log2FC) between the treatment groups, while the y-axis represents the negative logarithm of the p-value (-log10 p-value) for each gene. Each data point represents a gene, and differentially expressed genes (DEGs) are highlighted in red. The plot provides a visual representation of the distribution and significance of DEGs, with genes exhibiting higher fold change and statistical significance appearing farther away from the center of the plot.


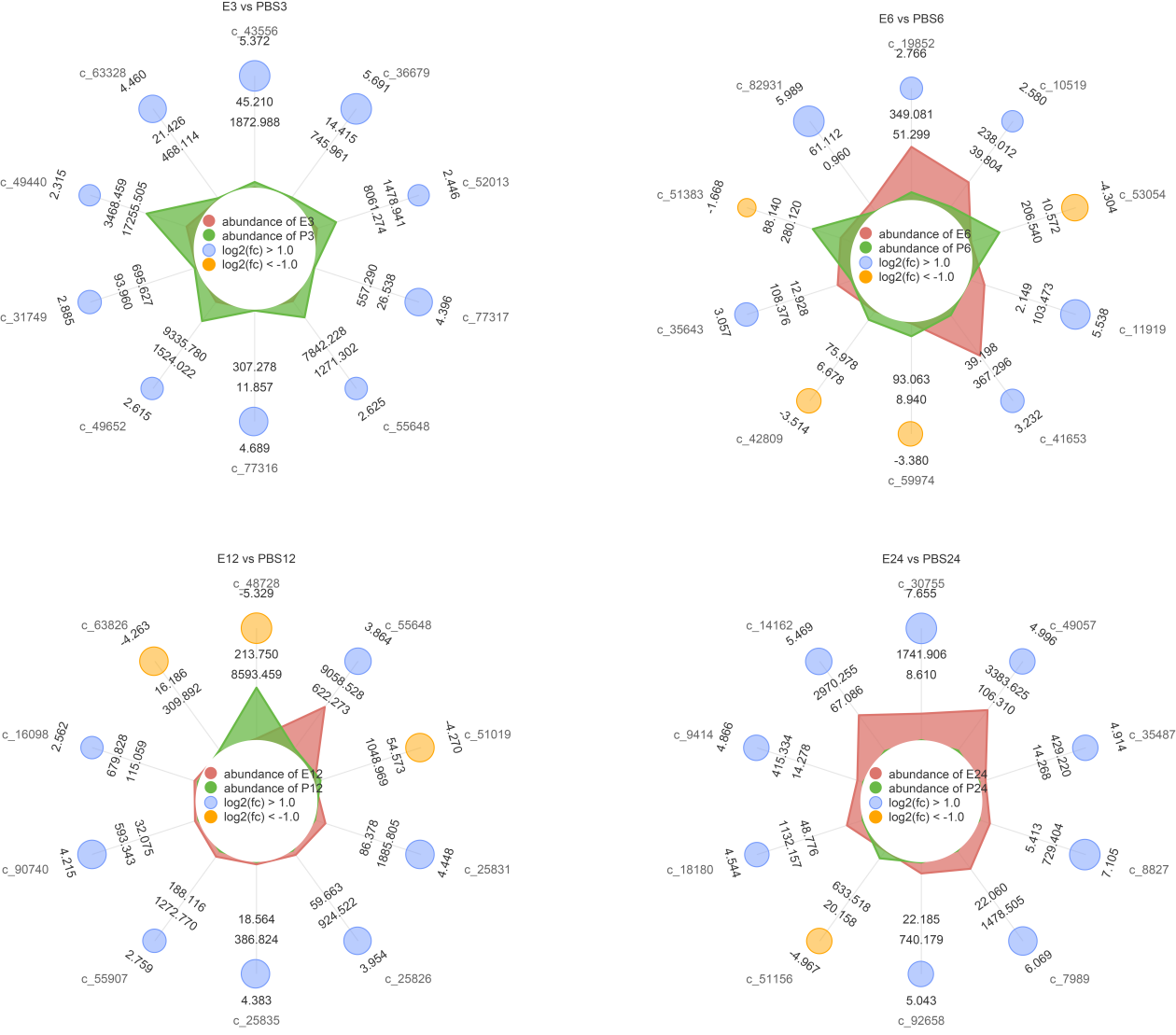


**Figure S6. Radar map illustrating the top ten altered DEGs in four comparisons.** Each spoke on the radar chart corresponds to a specific DEG, and the length of the spoke indicates the expression level. Different treatment groups are depicted by different colored lines. The radar map provides a visual comparison of the expression patterns of the top ten DEGs across different conditions, highlighting the genes that exhibit the most significant changes in expression.


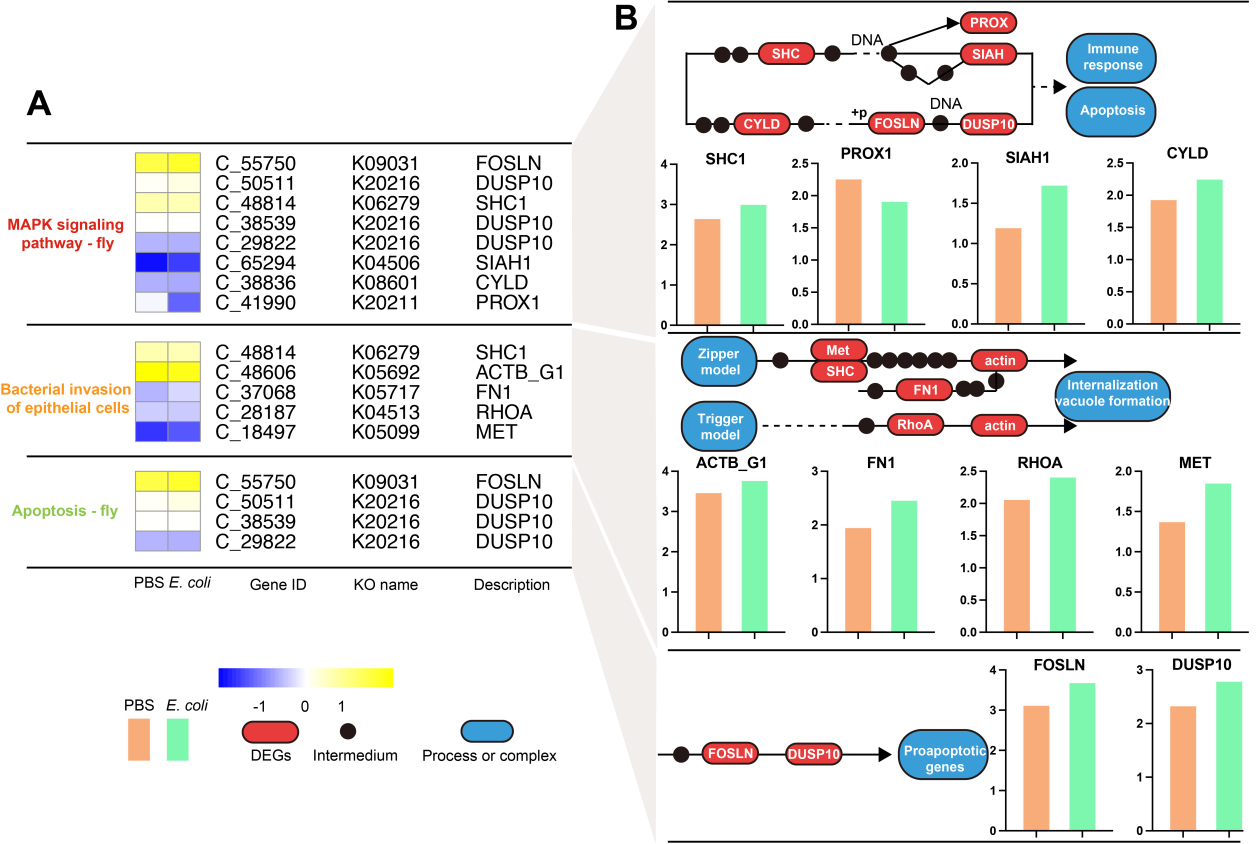


**Figure S7. Integrative analysis of differentially expressed genes (DEGs) using KO genes and KEGG modules within 3 h of *E. coli* stress.** (A) The relative expression levels of KO genes are associated with three pathways, including the MAPK signaling pathway, apoptosis, and bacterial invasion of epithelial cells. (B) Modules represent representative KO genes based on the classical KEGG pathway maps. The red box indicates an altered KO module (DEG), the dot represents an intermediate, and the blue rounded rectangle represents an indirectly altered biological process. The bar charts show the relative abundance of each KO module using log(readcount) values.


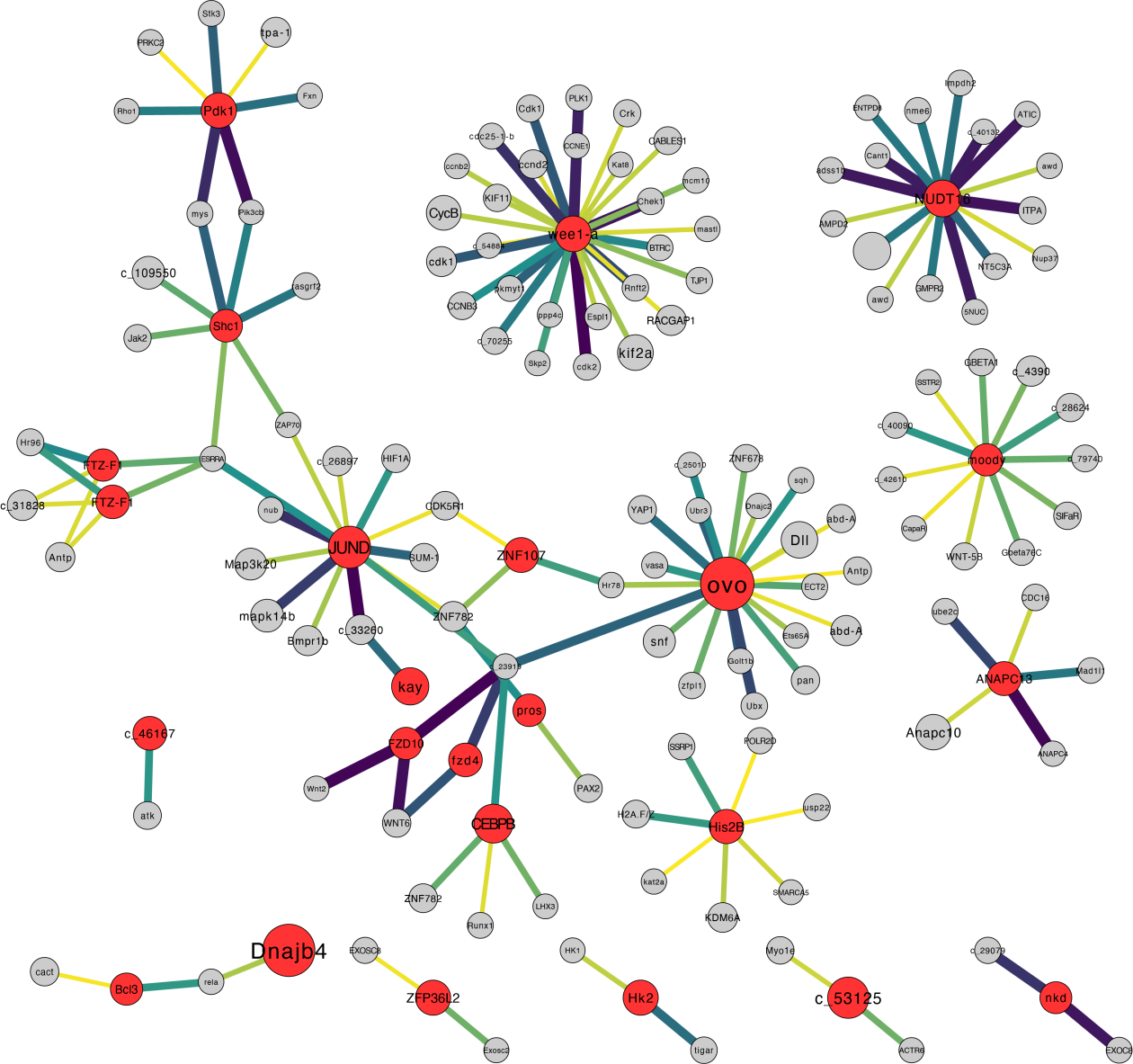


**Figure S8. Response of the protein interaction network in ticks within 3 h of *E. coli* stress.** The network of protein interactions and modular analysis. The diameters of the nodes reveal the relative amounts of gene expression. The edges depict the patterns of associations between particular genes.


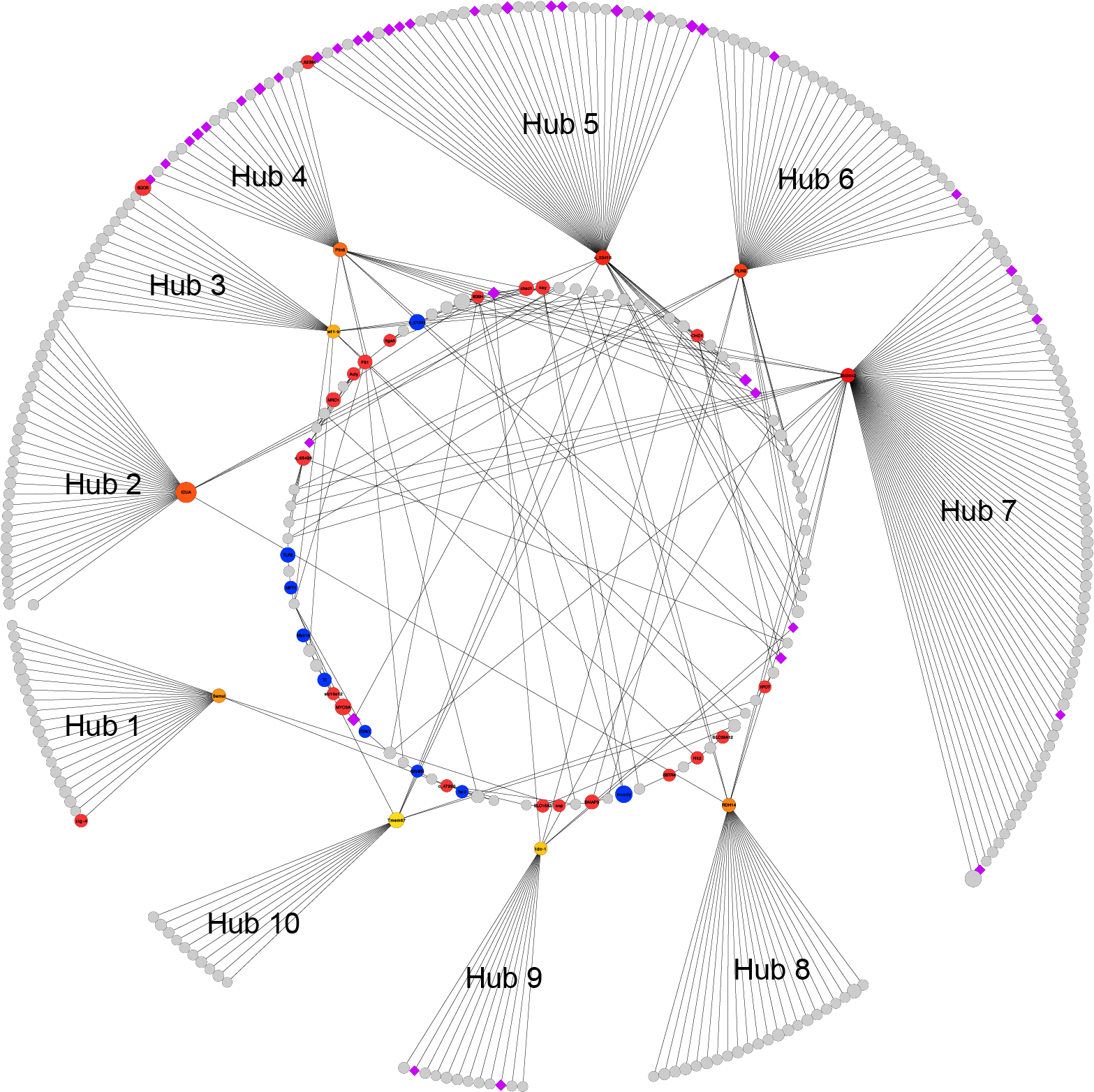


**Figure S9. Ten hub gene analysis using Cytoscape cytoHubba with the Maximum Clique Centrality (MCC) method.** The analysis identifies the top ten hub genes in the protein interaction network using the MCC method implemented in cytoHubba, a Cytoscape plugin. The hub genes are represented as nodes in the network, and their size indicates their centrality in the network. The MCC method determines the maximum clique centrality of each gene, reflecting its importance in maintaining network connectivity. The identified hub genes play crucial roles in the network and may serve as key regulators or interactors in the biological processes under investigation.


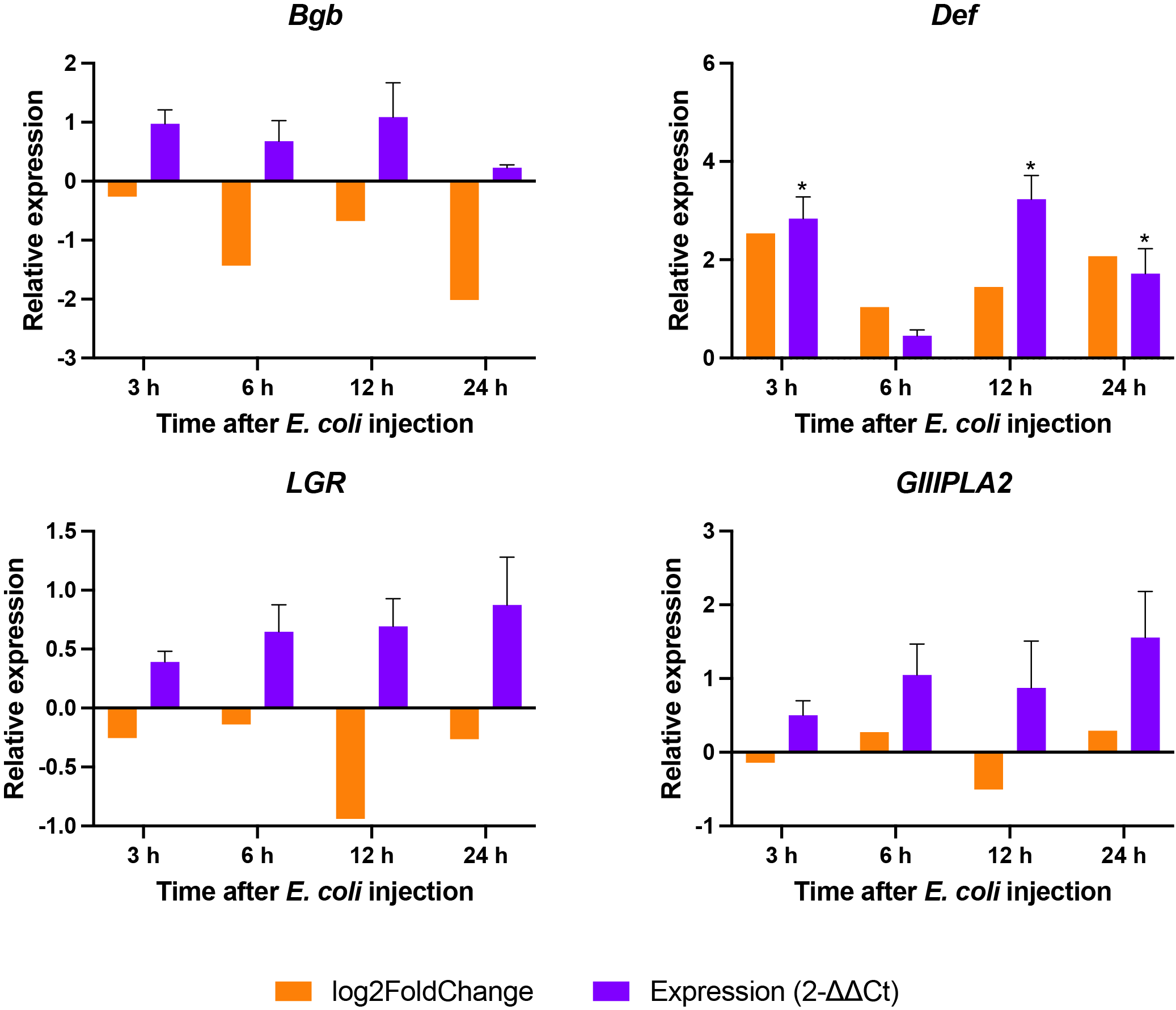


**Figure s10. Expression analysis and qRT-PCR verification of transcriptome data.** The fold change values and corresponding functions of the genes were obtained from the transcriptome analysis and are listed in Table S1. * indicates *Def* (defensin) is a DEG and the expression levels were significant compared with control group (*p* < 0.05).
